# Data article “Explaining the cyclical volatility of consumer debt risk using a heterogeneous agents model: The case of Chile”

**DOI:** 10.1016/j.dib.2019.103915

**Published:** 2019-05-30

**Authors:** Carlos Madeira

**Affiliations:** Central Bank of Chile, Agustinas 1180, Santiago, Chile

**Keywords:** Credit supply, Consumer credit, Default risk, Business cycle fluctuations, Unemployment shocks

## Abstract

This article provides data on the simulation results of consumer debt default for bank and non-bank lenders in Chile, using the model described in Ref. [1]. Furthermore, it provides a summary description of all the codes used for the simulation exercises and how to implement them from publicly available microdata sources. The data is of particular interest for those interested in analyzing the sensitivity of consumer loan default to heterogeneous labor market shocks and aggregate interest rates. All the codes and datasets are in Stata format.

**Table:** Data specifications

Subject area	Economics and Finance.
More specific subject area	Household Finance.
Type of data	Numerical data. Data files in Stata format (.dta), plus codes (Stata do-files).
How data was acquired	Data is output of implemented model (see Ref. [1], which was calibrated using publicly available survey datasets (EFH, EPF, ENE/ESI).
Software used	Stata MP-6 (version 13.1).
Data format	Floating numbers: %9.0g (right-justified, width 9, general number).
Experimental factors	Data is derived from microdata of household surveys (Finance, Employment, Expenditure) plus aggregate interest rates and consumer debt growth.
Experimental features	Final dataset is obtained from model simulation of relative to unemployment, interest rate and credit supply shocks.
Data source location	Chile, Santiago, Central Bank of Chile and INE (Institute of National Statistics).
Data accessibility	Data is available with this article.
Related Research Article	Madeira, C. (2018), “Explaining the cyclical volatility of consumer debt risk using a heterogeneous agents model: the case of Chile,” *Journal of Financial Stability*, 39, 209–220.

**Table:** 

**Value of the data** • The data can be used to analyze the sensitivity of the rates Non-Performing Loans (NPL) and Expenses with Non-Performing Loans (ENPL) to labor income and unemployment shocks experienced by different types of workers during the business cycle. Results can be separated according to lender type (banks or retail stores) and according to borrower's types (quintile of household income, age, education) for the entire period between 1990 and 2012 with a quarterly frequency. • Financial policy decision makers can observe a set of counterfactual stress scenarios and how households and lenders would suffer facing unpredictable negative events. The stress scenarios include higher interest rates (cost of credit shock), lower loan maturities (liquidity shock) and lower ceilings for the household credit lines (credit access shock). • The model includes a welfare cost of the financial and economic distress of the households, which is given by the consumption reduction experienced by households in each stress scenario. • Finally, the model and the data can be used by economic and financial researchers interested in the complexity and linkages between the household sector and aggregate risks in the economy [2], [3], [4], [6].

## 1 Data

Data files (.dta) and codes (.do) are in Stata 13 format. In the folder “Main_data_for_figures” the interested researchers can find the code “M_Cdebt_Graphs.do” which calls 10 datasets to create Table 6 plus Figures 1, 3, 4, 5, 6, 7, 8 and 9 in the original article of [1].

“Fig 1_delinquency_rates_countries.dta” contains the delinquency rates for Chile, Spain and the USA.

“Fig 1_delinquency_DIR_InterestRates_Unemployment_Chile.dta” contains the rates for Chile (in log differences over the mean) for the Consumer Delinquency, Debt to Income, Interest Rate and Unemployment.

“Fig 3_Simulated_NPL_ENPL_BankingSystem_1990_to_2012Quarterly.dta” contains the simulated and real NPL and ENPL rates for the Chilean banking system.

“Fig 4_Simulated_NPL_ENPL_NonBanks_1990_to_2012Quarterly.dta” contains the simulated NPL and ENPL rates for the Chilean Non Bank Lenders.

“Fig 4_Simulated_NPL_ENPL_AllLenders_1990_to_2012Quarterly.dta” contains the simulated NPL and ENPL rates for all Chilean Lenders.

“Fig 5_ConsumptionCost_IncomeHouseholdWeights.dta” shows the consumption reduction that financially stressed households suffer as a percentage of the average household income and as a percentage of the income for the entire economy.

“Fig 6_Simulated_NPL_ENPL_Banks_Quintiles_1990_2012.dta” contains the simulated NPL and ENPL rates by household income quintile (with quintile 1 being the 20% poorest households and 5 being the 20% richest households) for the Chilean Bank borrowers.

“Fig 7_Simulated_NPL_ENPL_NonBanks_Quintiles_1990_2012.dta” contains the simulated NPL and ENPL rates by household income quintile (with quintile 1 being the 20% poorest households and 5 being the 20% richest households) for the Chilean Non Bank borrowers.

“Fig 8_Simulated_NPL_ENPL_Banks_RiskScenarios_1990_2012.dta” contains the simulated NPL and ENPL rates for the Chilean Bank borrowers across 4 stress scenarios.

“Fig 9_Simulated_NPL_ENPL_NonBanks_RiskScenarios_1990_2012.dta” contains the simulated NPL and ENPL rates for the Chilean Non Bank borrowers across 4 stress scenarios.

## 2 Experimental design, materials and methods

### 2.1 Experimental design

The data consists of simulations of the Non-Performing Loans (NPL) and Expenses with Non-Performing Loans (ENPL) rates for Chilean households, using a structural model where households use behavioral rules to decide between consumption and defaulting on their loan commitments [1]. Lenders (banks or retail stores) offer a menu of contracts according to the risk of households and banks' funding costs, with loans differing in terms of interest rates, maturity and the debt amount available. Loan default simulations were then made for all the labor market shocks experienced during the period 1990 to 2012, including unemployment, permanent income and temporary income fluctuations (see Ref. [5]. The model then applied liquidity shocks in terms of the banks’ real funding costs and the maximum legal interest rate. Table 1 below provides a summary of the calibrated model used to create the final data from its source materials. See Section 3 and Section 4 in Ref. [1] for details.

**Table 1:** Calibrated and estimated parameters.

Parameters and Exogenous Shocks	Source
Population distribution and endowments	EFH 2007–2011
(Income, assets, debts |ζ)	ζ={Region, Sex, Age, Education, Industry, Quintile($Y_{t}$), Number of household Members}
Shocks to Initial Debt Endowments	Mean Debt and Interests Growth (SBIF)
Income dynamic shocks (540 types)	$Y_{t},\sigma_{t}$[5]; ENE 1990–2012)
Expenditure choice	$C_{t}=c\left(\zeta ,P_{t},\sigma_{t},\varepsilon^{c}\right)$ (EPF 2007) $m\left(\zeta \right)=Q_{1}\left(C_{0}|\zeta \right)$, λ=0.15
Default decisions	Budget kink: $\;B\left(g\left(\zeta ,C_{t+s-1},S_{t+s-1}\right)\right)<0$
Credit Market, 2 lenders (v=1,2)	Banks, Retail: $D_{v,t+1}\leq dc_{v,t+1}($lender v)
Loan terms: $i_{v,t}=i\left(.|CF_{t},X_{v,t}\right)$	EFH: $X_{v,t}=\left\{\zeta ,D_{t},P_{t},Y_{t},\text{Pr}\left(U_{t}\right),DS_{t}\right\}$
$m_{t}=\left\{m_{1,t},m_{2,t}\right\}$	$m_{t}=\left\{8,4\right\}$ (EFH data, MMFS, 2011)
$dc_{t}=\left\{dc_{1,t},dc_{2,t}\right\}$	$\{dc_{1}\left(P_{t},Y_{t},\zeta \right)$, $dc_{2}\left(P_{t},\zeta \right)\}$
Maximum Legal Interest Rate	$i_{v,t}\leq 1.50\times E\left[i_{2,t}\right]$
Banks' fundraising real interest rates, $i_{t}$	Central Bank of Chile (1990Q1-2012Q4)

### 2.2 Materials

The folder “SimulationCodes” contains all the simulation codes and algorithms that create the 10 datasets listed above from the original source data. See the file “Simulation Codes Summary.docx” for a brief explanation of all codes.

The model is calibrated using several sources of publicly available microdata, including the Chilean Household Finance Survey (EFH, waves of 2007–2011), the Employment Survey (ENE, waves of 1990-Q1 to 2012-Q4), and the Household Expenditure Survey (EPF, wave of 2007). Users can apply for all the waves of the Chilean Household Finance Survey (Encuesta Financiera de Hogares, EFH, waves of 2007–2011) by filling a form on the website of the Central Bank of Chile: http://www.bcentral.cl. The Employment Survey (Encuesta Nacional de Empleo, ENE), plus its Income Module (Encuesta Suplementaria de Ingresos), and the Household Expenditure Survey (Encuesta de Presupuestos Familiares, EPF, wave of 2007) are available from the website of the Chilean Institute of National Statistics (Instituto Nacional de Estadísticas, INE): http://www.ine.cl/ene/base-de-datos-ene.php; http://www.ine.cl/epf/VII/base-de-datos.php.

Also, some users can opt to calibrate the model by using the Chilean Income and Participation survey (Encuesta de Caracterización Socioeconómica Nacional, CASEN, wave of 2006), since the codes are prepared to do this automatically. The CASEN 2006 has less detailed financial information than the EFH, but it has a similar measure of income and a much larger sample (over 50,000 households): http://observatorio.ministeriodesarrollosocial.gob.cl/casen/casen_usuarios.php.

### 2.3 Methods

The sequence for the final data creation (which was simulated for the entire period between 1990 and 2012 at a quarterly frequency) can be described as follows:

1) The EFH dataset (waves 2007 to 2011) is used as the original 12,264 households' sample and households were randomly selected with replacement to form a 135,000 household population before the statistical simulation process.
2) Each of the labor force members of every household had a sequence of dynamic labor earnings' simulated with an industry earnings’ drift increase, plus idiosyncratic permanent and temporary wage shocks and flows into and out of unemployment [1]. This simulation sequence was implemented for the entire period between 1990 and 2012, with a quarterly frequency, using parameters estimated by Ref. [5].
3) Expenditure and consumption decisions by the households were calibrated using the EPF dataset (wave 2007) and a semi-parametric model that is linear on the log of the household's permanent income plus a continuous flexible function of its demographic characteristics (which includes home-ownership, employment status and age of the household head, Metropolitan Area, number of adults, minors, and senior members in the family).
4) Access to new loans from the credit market was calibrated using risk-adjusted interest rates (adequate for a competitive lender market) with a loan delinquency model estimated from the EFH dataset (2007–2011) with a probit function, using current income (in log), the debt over permanent income ratio, the debt service over current income ratio, the households' weighted unemployment risk, and demographic risk factors (including the age, education, gender, marriage status, region and county of the household head). A Maximum Legal Interest Rate and debt ceilings based on permanent income were applied for each households’ loan access decision [1].
5) Finally, the default decisions were calibrated based on the households' inability to keep both debt commitments and required expenditures and consumption within their budget constraint. The structural equations used for this decision and its budget constraint are detailed in Ref. [1].
6) 50 Bootstrap replicas of this procedure were repeated to calculate standard-errors and other dispersion statistics.
