# Supplementary material for: Data article “Explaining the cyclical volatility of consumer debt risk using a heterogeneous agents model: The case of Chile”
Source: Data Brief. 2019 May 30;25:103915. doi: 10.1016/j.dib.2019.103915 (PMC6557751; doi:10.1016/j.dib.2019.103915)
Supplement: Multimedia component 1 [file mmc1.docx]

I received no funding from any institution besides my employer which is the Central Bank of Chile. I have no conflicts of interest in the scientific and quantitative analysis of the consumer loan market in Chile.

Carlos Madeira 2^nd^ April 2019
