# Supplementary material for: Data article “Explaining the cyclical volatility of consumer debt risk using a heterogeneous agents model: The case of Chile”
Source: Data Brief. 2019 May 30;25:103915. doi: 10.1016/j.dib.2019.103915 (PMC6557751; doi:10.1016/j.dib.2019.103915)
Supplement: Multimedia component 2 [file mmc2.zip › SimulationCodes/Simulation Codes Summary.docx]

**Simulation Codes for the Article “Explaining the cyclical volatility of consumer debt risk using a heterogeneous agents model: the case of Chile”, *Journal of Financial Stability*, 39, 209-220.**

**Author:** Carlos Madeira

These codes use the original sources of data to create all the figures and analysis in the article. The Bootstrap codes create bootstrap replicas of the original dataset and 200 simulations can take around one week of computing time with Stata MP-6 in an ordinary notebook with 16 GB of Ram.

**Set of Codes 1):** See folder "figures". These codes replicate the final level of analysis, that is, the graphical outcomes.

M_Cdebt_System.do - This is the Master file that replicates all the figures in the article of Madeira (2018), with the exception of Figure 2. Associated sub-codes are: 1) debt_pir_evolution.do (creates Figure 1), 2) consumption_graphs.do (creates Figure 5 and Table 8), 3) CDebt_EFH_Banks.do (formats the data for use in Figure 3), 4) CDebt_Graphs_Banks.do (creates Figure 3), 5) CDebt_Graphs_NonBanks_All.do (creates Figure 4), 6) cons_risk_allscenarios.do (creates Figures 6 and 7 of default in banks and non-banks by quintile, plus Figures 8 and 9 of default in banks and non-banks according to risk scenarios in baseline, higher interest rates, lower loan maturities and lower debt ceilings).

**Set of Codes 2):** See folder "model_simulation". These codes apply the simulation model to the matched microdataset of all the surveys (EFH and/or CASEN - Household Finance Surveys; ENE - Employment and Income Surveys, formatted as in Madeira (2015); and EPF - Expenditure Survey) and then repeat the simulation for each set of labor market and credit shocks over the period 1990 to 2012 (quarterly frequency - therefore 92 time periods).

layoff_jobfind_yr.do (implements transitions between employment and unemployment in section 4.2 of the article), p_income_yr.do (implements permanent income in section 4.2 of the article), import_esi_stats.do (imports income growth and income volatility shocks estimated in Madeira, 2015), simulate_esi_income.do (simulates the dynamic income process described in section 4.2 of the paper, with income volatility and employment transitions), simulate_debt_ds_rci.do (adjusts the loan amount, debt service, and debt service to income ratio, after the aggregate adjustment described in footnote 4 of the article (Madeira, 2018), and the new dynamic income is simulated), simulate_default_pr.do (after the new debt is adjusted and the new income is simulated, this computes the 30 days in arrears default measure described in equation 11 of the article for a lender with full information), simulate_periods.do (this code simulates the new dynamics of loan indebtedness, income and consumption described in equations 2.1, 2.2, 3, 4.1 and 4.2 of the article, with an horizon of 8 quarters - it calls the following subroutines: adjust_consumption.do, allvars_default_pr.do, amortization_per.do, cupon_rates.do, debt_ceiling_per.do or in alternative debt_ceiling_per2.do, default_per.do, emp_trans_per.do, income_per.do, income_sh_yr2.do, ind_i_rates_per.do, layoff_jobfind_yr2.do, next_per_stats.do, saving_sticky_per.do).

**Set of Codes 3):** See folder "EFH_CASEN_EPF_format". These codes format the original microdata of all the surveys: EFH and/or CASEN - Household Finance Surveys; ENE - Employment and Income Surveys, from Madeira (2015); and EPF - Expenditure Survey.

all_scenarios.do (this code repeats the Master file for each one of the scenarios described in Figures 8 and 9 - ), hist_dynamic_un_households0 (this is the Master file that calls all the other codes and routines in Set of codes 2), 3) and 5), simul_format_efh3_0.do (this code formats all the EFH, CASEN and EPF, then matches them together in a single file of households which will be simulated over the period 1990 to 2012; the code also creates Figure 2 in the paper), simul_format_efh2_t.do (with t going from 07 to 11 - these codes format the EFH waves of 2007, 2008, 2009, 2010 and 2011, respectively), format_casen2006.do (formats the CASEN 2006 data), layoff_jobfind0.do (creates for each household the household weighted statistics for the risk of layoff in 3 months or the probability of regaining a job after 3 months), income_shock0.do (creates for each household a measure of the income volatility), p_income.do (creates each household’s permanent income by weighting each member’s income with their probabilities of employment and unemployment), replicate_data.do (each household is replicated a certain number of times - in this case 10 times - in order to reduce simulation error), format_epf.do (formats EPF 2007 data), epf_pincome.do (creates permanent income for each EPF household), epf_equivalence_c.do (creates percentiles for total expenditure, non-durables and durable goods in the EPF), simulate_consumption.do (simulates the model of equation 9 for the EFH/CASEN samples).

**Set of Codes 4):** See folder "bstrap_codes". These codes repeat all the steps in 2) and 3) for a pre-specified number of bootstrap replicas. The analysis code replicates Table 7 in Madeira (2018), but due to the large size of all the bootstrap replicas those datasets are only provided by the author upon request (note: 50 bootstrap replicas creates data files that are around 50 times as large as the original ones).

bstrap_default2_analysis.do (creates Table 7 in the article after all the bootstrap simulations are completed), bstrap_default2.do (makes the whole process of Set of Codes 2) and 3) for a number of pre-specified bootstrap replicas), hist_dynamic_un_households.do (repeats the master file of simulations over the period 1990 to 2012 for each bootstrap replica), bstrap_ene_esi_saved.do (makes bootstrap replicas of the ENE Employment Survey and its income module, the ESI), format_esi_epf_efh.do (it formats the EPF and EFH bootstrap replicas and joins them with the ENE/ESI bootstrap replica results), layoff_jobfind.do (bootstrap version that creates all_scenarios of the risk of layoff and the probability of jobfind), income_shock.do (bootstrap version that creates the household weighted measure of income volatility).

**Set of Codes 5):** See folder "algorithms". Short algorithms that help with repeated routines.

pctile_wgts.do or pctile_wgts2.do (do the same routine in different parts of the code - the algorithm calculates the percentiles of a variable y conditional on another categorical variable x and the population weights of the survey); mean_wgts.do or mean_wgts2.do (do the same routine in different parts of the code - the algorithm calculates the mean of a variable y conditional on another categorical variable x and the population weights of the survey); predict_xb_sd.do, predict_xb_sd2.do, predict_xb_sd2_facs.do, predict_xb_sd3.do (these codes do a similar routine in different parts of the code - they calculate a log-linear prediction of the mean and standard-deviation of a variable y); linear_reg_impute3.do (when data is missing this code makes an imputed value from a linear model plus a random error term, using coefficients computed from the algorithms predict_xb_sd.do, predict_xb_sd2.do, predict_xb_sd2_facs.do, predict_xb_sd3.do).
